# The 2024 NIA-AA biological definition of Alzheimer’s disease: linking biomarkers to clinical practice

**DOI:** 10.3389/frdem.2026.1736297

**Published:** 2026-02-11

**Authors:** Goh Kobayashi, Kosei Hirata, Maiko Ono, Kensaku Kasuga, Yuhei Takado

**Affiliations:** 1 Institute for Quantum Life Science, National Institutes for Quantum Science and Technology, Chiba, Japan; 2 Icahn School of Medicine at Mount Sinai, New York, NY, United States; 3 Department of Diagnostic Innovation Science, National Center for Geriatrics and Gerontology, Obu, Japan

**Keywords:** A/T/N framework, Alzheimer’s disease, amyloid PET, co-pathologies, fluid biomarkers, NIA-AA 2024 diagnostic criteria, resilience, tau PET

## Abstract

The 2024 National Institute on Aging–Alzheimer’s Association (NIA-AA) criteria establish a biological definition of Alzheimer’s disease (AD), marking a pivotal step toward linking research biomarkers with clinical practice. This review traces the evolution of AD diagnostic frameworks from the 1984 NINCDS-ADRDA clinical criteria, through biomarker-informed updates in 2011, to the 2024 biology-based criteria that bridge research and clinical care. The 2024 framework defines AD by its underlying pathology rather than clinical symptoms, recognizing that biomarker evidence alone can establish diagnosis. It expands the traditional AT (N) model into a multimodal profile (AT_1_T_2_NISV), in which Core-1 biomarkers (A and T_1_) are diagnostic, while Core-2 biomarkers (T_2_) support biological staging. Non-specific but mechanistically important processes (N, neurodegeneration; I, inflammation) and common co-pathologies (S, *α*-synuclein; V, vascular injury) are also incorporated to better capture the complexity of late-life dementia. Recent advances in plasma and PET biomarkers, including p-tau217, mid-region p-tau, and *α*-synuclein imaging, are redefining biological diagnosis and expanding its reach. Moreover, co-pathologies involving TDP-43, glial dysfunction, and vascular factors contribute to disease heterogeneity and variable therapeutic response. While the 2024 criteria represent a major conceptual step forward, they should be regarded as a dynamic framework open to future integration of emerging biomarkers. Bridging molecular pathology, neuroimaging, and clinical presentation will be essential to realize the goal of patient-centered precision medicine in AD. In this review, we synthesize recent advances in biomarker-based frameworks for AD and discuss co-pathologies, resilience-related modifiers, and emerging evidence challenging traditional interpretations of structural neurodegeneration markers. We also address implications for clinical implementation, including PET standardization and disease-modifying therapies.

## 1 Introduction

Alzheimer’s disease (AD) is neuropathologically defined and diagnosed by the co-existence of amyloid-*β* (Aβ) plaques and tau neurofibrillary tangles (NFTs) (Hyman et al., 2012). Amyloid plaques are formed by the deposition of amyloid-*β* (Aβ) in the extracellular space, whereas NFTs are formed by the intracellular accumulation of hyperphosphorylated tau. The physiological function of Aβ has not been fully elucidated, however, tau is known to bind to microtubules and regulate axonal transport (Gendron and Petrucelli, 2009; Bernabeu-Zornoza et al., 2019). Cases with amyloid plaque positivity but without NFTs are referred to as Alzheimer’s disease neuropathologic change (ADNC), whereas cases with tau tangles in the absence of amyloid plaques are classified as non-AD tauopathies (Hyman et al., 2012).

Beyond neuropathologic diagnosis, AD is now recognized as a biological continuum that progresses temporally from normal cognition through mild cognitive impairment (MCI) to dementia, characterized by sequential amyloid and tau pathology (Jack et al., 2018). With the development of AD biomarkers, it has become possible to evaluate the underlying pathophysiology *in vivo*, and in 2010 a hypothetical model was proposed suggesting that AD follows a temporal evolution linking biomarker changes to clinical symptoms; this concept has since been refined through subsequent revisions (Jack et al., 2010, 2013). This model revealed that Aβ accumulation begins more than a decade before the onset of cognitive impairment, emphasizing the need to define AD biologically rather than symptomatically (Jagust and Landau, 2021). Within this framework, the AT(N) classification system was proposed, enabling individuals along the Alzheimer’s continuum to be categorized according to their biological biomarker profiles (Jack et al., 2016).

## 2 Evolution of biomarkers and Alzheimer’s disease diagnostic criteria

**Figure 1:**
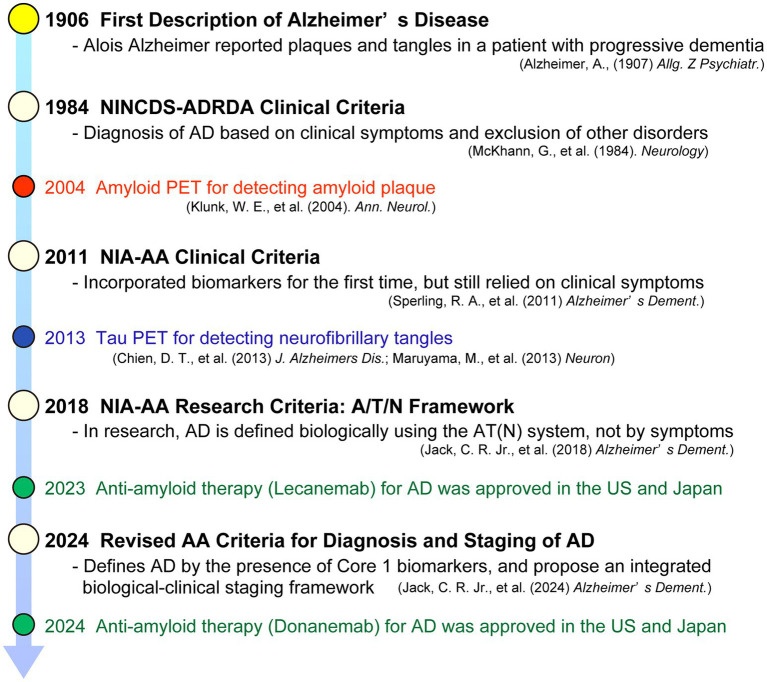
Chronological evolution of Alzheimer’s disease diagnostic criteria and biomarkers (1906–2024). Timeline illustrating the conceptual and technological milestones in the diagnosis and biological definition of Alzheimer’s disease. Color-coded circles indicate the nature of each milestone: red, amyloid PET imaging; blue, tau PET imaging; green, disease-modifying anti-amyloid therapies; and white, diagnostic or conceptual frameworks. Representative amyloid PET tracers include ^11^C-PiB, while representative tau PET tracers include ^18^F-flortaucipir and ^11^C-PBB3. Key milestones are indicated by year, including the introduction of biomarker-informed diagnostic criteria in 2011, the AT (N) research framework in 2018, and the revised NIA-AA biological definition and staging framework in 2024.

This chapter traces the field’s evolution, in parallel with advances in biomarkers, from the 1984 NINCDS–ADRDA clinical-only criteria to the 2011 NIA–AA biomarker-informed updates, the 2018 research framework that biologically defines AD, and finally the National Institute on Aging–Alzheimer’s Association (NIA-AA) 2024 criteria that introduce biological staging and bridge research and clinical contexts. This chronological development is summarized in Figure 1, which highlights major milestones in the evolution of AD diagnostic criteria and biomarker discovery—from the clinicopathologic era to the current biologically based definition.

### 2.1 1984: clinical-only criteria (NINCDS-ADRDA)

Before the development of biomarkers, the 1984 NINCDS–ADRDA criteria clearly stated that “Diagnosis cannot be determined by laboratory tests,” and the diagnosis of AD relied entirely on clinical manifestations and the exclusion of other causes of dementia (McKhann et al., 1984). This framework reflected the limited understanding of AD pathophysiology at the time when *in vivo* markers of amyloid and tau pathology were not yet available.

### 2.2 2004: emergence of amyloid PET imaging

In 2004, amyloid PET emerged as a groundbreaking imaging technology that enabled in vivo visualization of amyloid plaques and revolutionized AD research (Klunk et al., 2004). Among AD biomarkers, amyloid appears earliest and serves as an essential marker for diagnosis. However, the rate of Aβ deposition slows only a few years after reaching the positivity threshold and reaches a plateau by the MCI stage, making it challenging to use as a staging or monitoring marker (Jagust and Landau, 2021).

The first radiotracer developed, ^11^C-labeled Pittsburgh compound B (^11^C-PiB), binds with high sensitivity and specificity to fibrillar Aβ aggregates. A major limitation, however, is the short 20-min half-life of the ^11^C isotope, restricting its use to research PET centers equipped with an on-site cyclotron. To overcome this, several ^18^F-labeled tracers with a longer half-life (−110 min) were developed, and ^18^F amyloid PET is now approved and used worldwide (Rabinovici et al., 2025).

### 2.3 2013: development of tau PET

Following the success of amyloid PET, tau PET capable of detecting NFTs was developed in 2013, marking another major milestone in AD imaging (Chien et al., 2013; Maruyama et al., 2013). By combining tau and amyloid PET, *in vivo* visualization of the two cardinal pathologies that define AD became possible for the first time, enabling investigation of the spatial and temporal interplay between Aβ and tau deposition.

In 2020, ^18^F-flortaucipir (FTP) became the first tau-selective tracer to receive approval from the U. S. Food and Drug Administration (FDA) for estimating the distribution and density of NFTs in patients with cognitive impairment being evaluated for AD. Although several other tau-targeted tracers have been developed, FTP remains the only one approved for clinical use. All tau tracers bind selectively to paired helical filament tau typical of AD, showing minimal-to-weak binding in non-AD tauopathies (Groot et al., 2022). Quantitatively, tau PET can estimate Braak stages V–VI and high ADNC with excellent sensitivity (92–100%) and moderate-to-high specificity (50–92%) (Fleisher et al., 2020).

Beyond AD, advances in tau imaging have expanded its application to non-AD tauopathies. The tracer originally known as PM-PBB3—now termed florzolotau (^18^F)—was developed as an extension of the earlier work (Maruyama et al., 2013) and has demonstrated high-contrast detection of progressive supranuclear palsy (PSP) and corticobasal degeneration (CBD) tau pathology (Tagai et al., 2021). This pan-tau imaging capability represents an important step toward differential diagnosis among tauopathies.

### 2.4 Non-imaging biomarkers: CSF and plasma assays

Early studies in the 1990s, using sensitive sandwich ELISA methods, demonstrated that cerebrospinal fluid (CSF) total tau (t-tau)—a soluble N-terminal fragment derived from the axonal tau protein rather than the filament core of NFTs—was elevated in clinically diagnosed AD, whereas Aβ42, the principal peptide forming amyloid plaques, was decreased (Vandermeeren et al., 1993; Arai et al., 1995; Blennow et al., 1995; Motter et al., 1995). Initial work with phosphorylation-specific antibodies (e.g., AT8; Ser199–202) showed limited CSF reactivity, but subsequent assays targeting epitopes such as T181, S199, T205, T217, and T231 confirmed consistent increases in phosphorylated tau (p-tau) in AD (Xia et al., 2021; Gobom et al., 2022). These findings established CSF biomarkers as reliable tools for *in vivo* diagnosis of AD (Olsson et al., 2016; Iaccarino et al., 2023). Although lumbar puncture is generally safe and well tolerated, its perceived invasiveness, need for specialized staff, and contraindications (e.g., anticoagulant use) have limited broader use (Schindler and Atri, 2023). Consequently, attention has shifted toward blood-based biomarkers that are easier to collect and more scalable in clinical settings.

Early studies also reported increases in plasma Aβ in AD during the 1990s, but limited assay sensitivity and reproducibility hindered clinical application (Mehta et al., 2000; Hansson et al., 2010). This changed with the development of immunoprecipitation–mass spectrometry (IP-MS), which enabled accurate measurement of plasma Aβ42/40 ratios and demonstrated a strong correlation with abnormal brain amyloid burden (Ovod et al., 2017; Nakamura et al., 2018). Subsequently, plasma p-tau biomarkers showed strong concordance with amyloid and tau PET as well as CSF biomarkers, reflecting ADNC with high diagnostic accuracy (Janelidze et al., 2020; Karikari et al., 2020; Palmqvist et al., 2020; Thijssen et al., 2020; Janelidze et al., 2021). Among them, p-tau217 performs comparably to CSF measures in classifying amyloid and tau PET status, markedly increasing the clinical utility of blood-based biomarkers (Barthélemy et al., 2024).

### 2.5 2011: introduction of biomarkers into NIA-AA clinical criteria

Nearly three decades after the 1984 NINCDS–ADRDA criteria, the 2011 NIA–AA guidelines revised the clinical diagnostic framework for dementia due to AD (McKhann et al., 2011). The updated criteria remained primarily clinical, defining the dementia syndrome based on cognitive and functional findings. However, for the first time, CSF and amyloid PET biomarkers were incorporated as supportive evidence of the underlying pathophysiology, although routine biomarker testing was not yet recommended for clinical use. Structural MRI markers of neurodegeneration, including medial temporal lobe atrophy, were also recognized as supportive features of AD-related neurodegeneration in research settings. In a landmark conceptual shift, companion articles proposed viewing AD as a continuum of clinical and biological processes (Albert et al., 2011; Sperling et al., 2011). Whereas the term Alzheimer’s disease had previously referred only to the dementia stage, the revised framework expanded the construct to include MCI due to AD and, for research purposes, asymptomatic individuals with positive amyloid or CSF biomarkers—termed preclinical AD. This redefinition established the foundation for the biological staging of AD that would later be formalized in the 2018 and 2024 NIA–AA criteria.

### 2.6 2018: establishment of the biological research framework

Driven by advances in biomarkers and by recognition of the limitations of symptom-based definitions in a disease that unfolds along a continuum, the 2018 NIA–AA research framework redefined AD in biological terms for research purposes (Jack et al., 2018). The framework proposed that “Alzheimer’s disease is defined by its underlying pathologic processes, which can be documented by postmortem examination or *in vivo* by biomarkers. The diagnosis is not based on the clinical consequences of the disease (i.e., symptoms or signs)”. Importantly, neurodegeneration markers, including structural MRI measures, were explicitly repositioned as indicators of disease severity rather than defining features of AD pathology. The framework had three main objectives: (i) to standardize research methodology, (ii) to refine participant selection for disease-modifying therapies (DMTs) trials, and (iii) to improve understanding of disease etiology. Following the AT(N) classification system, AD was defined biologically as the presence of both A (aggregated amyloid-*β* or its associated pathologic state: CSF Aβ42 or Aβ42/Aβ40 ratio, or amyloid PET) and T (aggregated tau (NFTs) or its associated pathologic state: CSF phosphorylated tau or tau PET) positivity. In the 2011 NIA–AA guidelines, CSF p-tau and t-tau had been regarded together as markers of neuronal injury. However, since p-tau rises more specifically in AD, whereas t-tau can be elevated in other neurological disorders, the 2018 framework designated p-tau as the T marker and t-tau as the N (neurodegeneration) marker. At that time, “p-tau” referred primarily to p-tau181, the most validated phospho-epitope. Because this biological definition had not yet been validated in large longitudinal cohorts or randomized clinical trials, the authors explicitly restricted its use to a research framework, emphasizing that “it is premature and inappropriate to apply this research framework in general medical practice”.

Over the following years, rapid progress in biomarker technology—especially the development of highly accurate plasma p-tau assays and tau PET imaging—narrowed the gap between research and clinical application. The approval of anti-amyloid therapies further underscored the need for standardized, biologically grounded diagnostic and staging criteria that could guide both care and research.

### 2.7 2024: transition to biology-based diagnosis in clinical care

The 2024 NIA–AA criteria provide a biology-based framework for the diagnosis and staging of AD, aiming to bridge research and clinical practice (Jack et al., 2024). The revision was undertaken for three main reasons: (i) with the approval of DMTs, standardized diagnostic and staging systems are needed across clinical, research, and industry settings; (ii) the emergence of blood-based biomarkers has made biological diagnosis increasingly accessible; and (iii) accumulating evidence supports the partial interchangeability of different biomarker modalities. The revised framework is grounded in several fundamental principles: AD is defined by its biology rather than by its symptoms; biomarker evidence of AD neuropathology is sufficient for diagnosis; AD exists on a continuum that begins asymptomatically and progresses biologically, with clinical symptoms emerging later but not required for diagnosis; and similar clinical syndromes may result from non-AD causes, meaning that symptoms alone are not diagnostic. Nonetheless, these criteria are not intended as clinical practice guidelines. Because biomarker testing is not universally accessible, the document is not designed as a step-by-step protocol for all care settings, nor does it prescribe specific treatment pathways.

### 2.8 Diagnostic structure in the 2024 criteria

#### 2.8.1 Biological diagnosis (Core-1)

In the 2024 NIA-AA criteria, AD can be diagnosed biologically on the basis of any single Core-1 biomarker positivity, specifically amyloid PET, CSF Aβ42/40, CSF p-tau181/Aβ42, CSF t-tau/Aβ42, or a validated plasma assay such as p-tau217. Diagnosis is thus defined biologically rather than clinically and can be made regardless of the presence or absence of symptoms. The criteria expand the traditional AT(N) classification system into a multimodal profile (AT_1_T_2_NISV). Disease specific Core biomarkers are divided into two groups: Core-1, comprising A (amyloid pathology) and T_1_ (soluble tau fragments), is used for biological diagnosis; Core-2, representing T_2_ (aggregated tau pathology detected by tau PET), serves primarily for biological staging. Additional dimensions, N (neurodegeneration) and I (inflammation), represent nonspecific but mechanistically important processes in AD pathogenesis, while S (*α*-synuclein) and V (vascular brain injury) denote common non-AD co-pathologies. In essence, AD can be diagnosed solely based on an amyloid biomarker (amyloid PET or CSF Aβ42/40 ratio). At first glance, this may seem to allow diagnosis on amyloid pathology alone, without evidence of tau pathology. However, this is not the case: the framework assumes that amyloid PET positivity implies the presence of both amyloid and tau pathology required for the neuropathologic diagnosis of AD. Specifically, visual interpretation of amyloid PET detects at least moderate neuritic plaques on the CERAD neuritic plaque score, a semiquantitative measure of plaque burden, with sensitivity of 86 to 98% and specificity of 89 to 100% (Clark et al., 2012; Curtis et al., 2015; Sabri et al., 2015; La Joie et al., 2019; Lowe et al., 2019). Among 4,637 autopsy cases with moderate or frequent neuritic plaques during life who showed cognitive impairment, 95% of cases had Braak NFT stage III or higher, a semiquantitative measure of tangle severity. Thus, the presence of moderate neuritic plaques generally implies coexisting tau pathology, satisfying the neuropathologic criteria for intermediate-to-high ADNC. Even among individuals with moderate neuritic plaque burden who were cognitively unimpaired during life, 74–87% of cases had Braak stage III or higher, and 91% had Braak stage II or higher (Beach et al., 2015). Moreover, in 34 cognitively normal, amyloid-PET-positive individuals who later progressed to dementia or MCI and underwent autopsy, 94% had intermediate- or high-likelihood ADNC (Roberts et al., 2008). Based on these findings, the authors proposed that “most cognitively unimpaired persons with an abnormal amyloid PET scan should presently meet neuropathologic criteria for intermediate/high ADNC, and those that do not will very likely do so in the future if they live long enough.” However, this high concordance between amyloid PET positivity and tau pathology has been primarily demonstrated in autopsy-based cohorts of older individuals, and may not be directly generalizable to younger populations or to cohorts with different ethnic or genetic backgrounds.

CSF Aβ42/40 ratio, p-tau181/Aβ42 ratio, and t-tau/Aβ42 ratio measured using FDA-approved in-vitro diagnostic assays identify amyloid-PET visual positives with sensitivity (positive percent agreement, PPA) of 85–92% and specificity (negative percent agreement, NPA) of 89–94%. Therefore, CSF biomarker positivity can likewise be interpreted as consistent with AD (U.S. Food and Drug Administration, 2022b, 2022a; Iaccarino et al., 2023). With accumulating evidence and regulatory progress, plasma p-tau217 now performs at CSF-like levels and has been included among Core-1 diagnostic options (Therriault et al., 2023a). Together, these observations indicate that the diagnostic criteria are grounded in multiple interrelated assumptions regarding the biological correspondence between *in vivo* biomarkers and neuropathologic change.

#### 2.8.2 T_1_ and T_2_ biomarkers

T biomarkers are subdivided into two categories. T_1_ refers to biofluid measures of phosphorylated and secreted tau species that become abnormal early in the disease, whereas T_2_ represents biomarkers of aggregated tau neurofibrillary pathology that become abnormal later. Converging evidence shows that secretion of tau fragments phosphorylated at specific residues such as p-tau181, p-tau217, and p-tau231 increases after Aβ pathology emerges and before tau PET becomes positive (Barthélemy et al., 2020; Mattsson-Carlgren et al., 2020; Janelidze et al., 2021; Therriault et al., 2023b). This early increase is thought to reflect a physiological response to Aβ plaque deposition and may link Aβ proteinopathy to the onset of tau proteinopathy (Sato et al., 2018; Mattsson-Carlgren et al., 2021; Pichet Binette et al., 2022). By contrast, other tau fragment analytes such as microtubule binding region tau (MTBR tau243) and non-phosphorylated mid-region tau fragments track tau PET signals more closely and tend to change later in the disease course (Horie et al., 2022; Salvadó et al., 2023). Consistent with these findings, a recently developed plasma assay detecting mid-region p-tau181 fragments—rather than conventional N-terminal epitopes—has been shown to correlate strongly with tau PET burden, independent of amyloid accumulation, and to reflect later-stage tangle pathology (Tagai et al., 2024). Although mid-p-tau181 is not yet included among the Core biomarkers defined in the 2024 NIA-AA criteria, its temporal pattern and pathological associations indicate that it aligns more closely with the T_2_ category, reflecting later-stage tau aggregation. Accordingly, A (amyloid) and T_1_ together constitute Core-1 biomarkers sufficient for biological diagnosis, whereas T_2_ serves primarily to support biological staging and prognostic assessment. Furthermore, t-tau, which was classified into the N category in the 2018 NIA-AA research framework, has been reclassified into the T category in the 2024 NIA-AA criteria. However, t-tau is a biomarker correlated with both p-tau and neurodegeneration. While it has been reported that t-tau correlates with both p-tau and NfL (Lewczuk et al., 2018; Jin et al., 2019), we previously reported that, within the AD continuum, CSF t-tau represents a pathological process distinct from that reflected by CSF NfL (Kasuga et al., 2023), suggesting that t-tau may not represent a unitary marker of neurodegeneration. Importantly, these findings do not contradict the conceptual foundation of the 2018 AT(N) framework, in which neurodegeneration (N) was intentionally defined as a staging marker rather than a core diagnostic component. Rather, they support the view that refinements such as T_1_/T_2_ stratification or alternative categorization of t-tau are hypothesis-generating and require longitudinal validation to clarify their temporal dynamics and clinical utility.

#### 2.8.3 Biological and clinical staging

**Figure 2:**
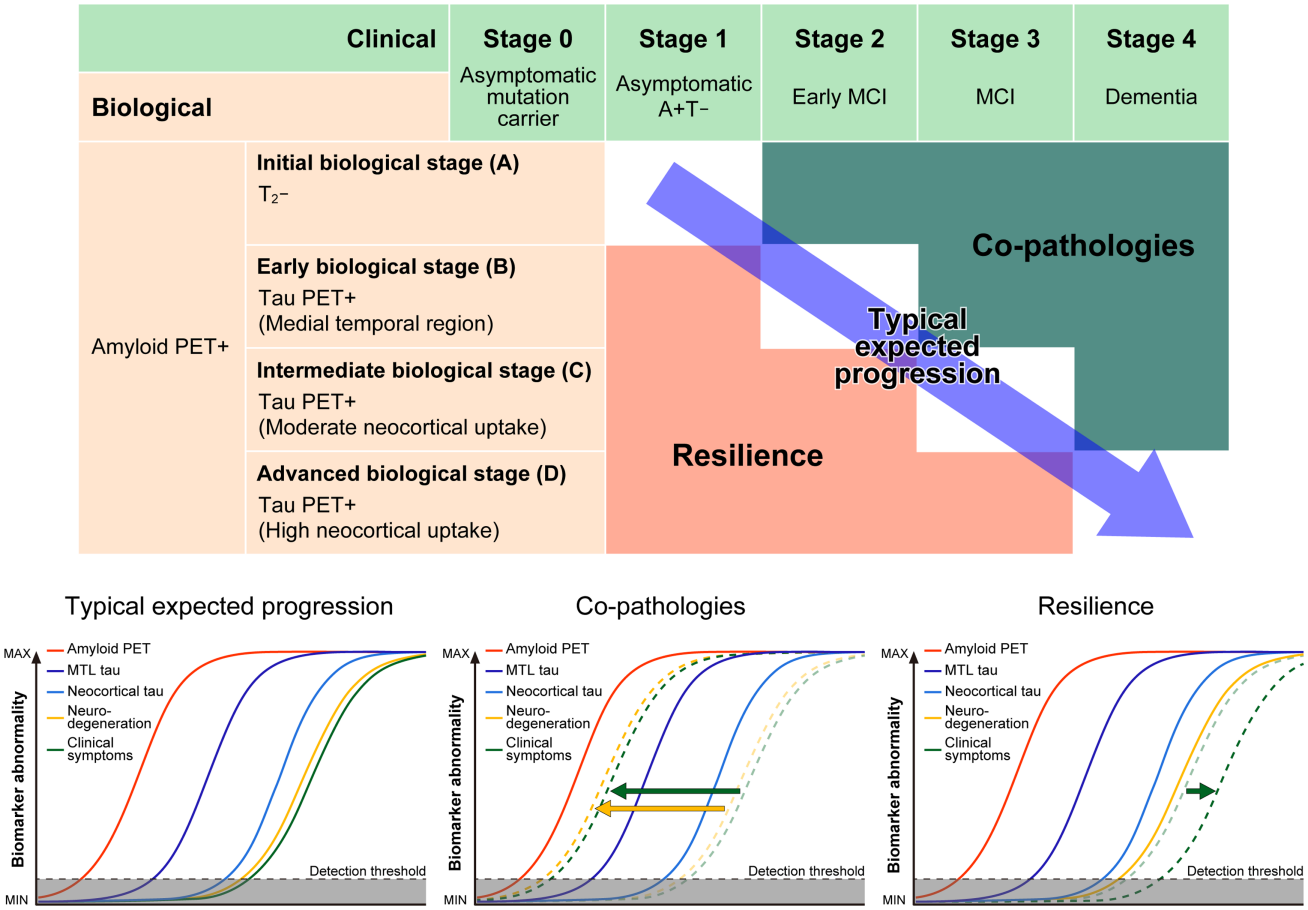
Conceptual framework linking biological and clinical staging in Alzheimer’s disease under the 2024 NIA–AA criteria. The upper panel illustrates the relationship between biological disease stage and clinical stage across the AD continuum. Biological stages are defined by amyloid and tau PET profiles, progressing from amyloid positivity without tau aggregation to increasing neocortical tau burden, while clinical stages range from asymptomatic to dementia. The diagonal arrow indicates the typical expected progression from early biological changes to clinical impairment. The shaded regions highlight modifying factors that alter this relationship. Co-pathologies, including vascular injury, α-synuclein pathology, and TDP-43–related processes, can accelerate clinical progression relative to biological stage. In contrast, resilience-related factors can delay the emergence of clinical symptoms despite comparable levels of underlying pathology. The lower panels schematically depict biomarker trajectories under three scenarios: typical expected progression, accelerated progression driven by co-pathologies, and delayed clinical expression associated with resilience. Together, the figure provides a graphical summary of how biological pathology, co-pathologies, and resilience interact to produce the clinical heterogeneity emphasized throughout this review. Figure modified from Jack et al. (2024), Alzheimer’s and Dementia, with adaptations and emphasis added for clarity.

In the 2018 research framework, to avoid confusion with clinical stages, the “plus/minus” combinations of AT(N) were used informally as biomarker profiles rather than formal stages. Although individuals within the Alzheimer’s continuum were expected to progress from A + T − N − to A + T + N − to A + T + N+, the AT(N) classification system did not specify a fixed order of events or imply causality (Jack et al., 2018). In contrast, the 2024 revision explicitly recognizes potential discrepancies between biology and clinical presentation and recommends an integrated scheme that incorporates a biological stage, a clinical stage, and their combination (Jack et al., 2024; Figure 2). Biological staging applies only to individuals diagnosed using Core-1 biomarkers and relies solely on Core biomarkers (A, T_1_, and T_2_). N biomarkers are excluded because “the temporal relationships between core AD biomarkers and N biomarkers are inconsistent across individuals.” Because imaging captures both topographic and quantitative information, amyloid PET and tau PET are adopted for biological staging. Indeed, the assigned stage can vary depending on the topographic information provided by tau PET. In clinical practice, since Core-1 fluid biomarkers may be obtained before anti-amyloid therapy, amyloid PET need not be performed when Core-1 fluid biomarkers are available. A fluid-only staging approach has also been proposed conceptually; however, PET and fluid measures are not equivalent. Therefore, stages A–D defined by PET should not be considered identical to stages A–D derived from fluid biomarkers. Recognizing that biology and clinical features do not progress in lockstep across all individuals, the revised framework aims to integrate biological and clinical staging. Discrepancies between biological and clinical stages are attributed to co-pathologies and resilience, which will be discussed later.

## 3 Co-pathologies in Alzheimer’s disease

Aging increases both the frequency and heterogeneity of neuropathological changes (Rahimi and Kovacs, 2014). In individuals with AD pathology, coexisting pathologies become more common and more severe as the disease progresses, and the extent of AD pathology correlates with that of these co-pathologies (Robinson et al., 2021). Clinically, the coexistence of multiple pathologies is associated with approximately a threefold higher odds of dementia (Schneider et al., 2007). Therefore, understanding non-AD pathologies that coexist with AD, collectively referred to as co-pathologies, is essential for elucidating the full biological spectrum of dementia. Importantly, recent advances in *in vivo* biomarker research have enabled the characterization of such co-pathologies, and the 2024 NIA-AA criteria, for the first time, formally include S (*α*-synuclein) and V (vascular brain injury) as biomarkers of non-AD co-pathologies. Although TDP-43–related pathology is not yet included as a biomarker category in the 2024 framework due to the current lack of validated in vivo markers, it is discussed in the report as an emerging and increasingly recognized contributor to late-life dementia. Therefore, the following section also addresses TDP-43–related pathology and its relevance within the broader co-pathology spectrum.

### 3.1 *α*-Synuclein in pathology

In α-synucleinopathies such as Parkinson’s disease (PD) and dementia with Lewy bodies (DLB), it has been proposed, analogous to AD, to define disease entities biologically rather than by clinical syndromes (Simuni et al., 2024). This paradigm shift was made possible by the advent of a robust biomarker, the CSF *α*-synuclein seed amplification assay (SAA), which exploits the prion like propagation of *α*-synuclein to enable biological classification (Fairfoul et al., 2016; Shahnawaz et al., 2017; Grossauer et al., 2023). The availability of such a biological marker has facilitated the identification of *α*-synuclein co-pathology within AD using multimodal biomarker profiles. Approximately half of AD cases reportedly harbor α-synuclein (Lewy body) pathology. Compared with “pure” AD, mixed AD/Lewy body cases more frequently present with DLB like clinical features and show faster cognitive decline and higher mortality than either pure AD or pure DLB (Thal et al., 2025). Physiologically, *α*-synuclein may interact with Alzheimer’s pathology to accelerate disease progression, potentially accounting for inter-individual variability in the clinical course (Gawor et al., 2024). Moreover, *α*-synucleinopathy inferred by SAA influences cognitive function independently of AD pathology, both cross sectionally and longitudinally, thereby contributing substantially to discrepancies between biological and clinical staging when present as a co-pathology (Palmqvist et al., 2023; Quadalti et al., 2023). Beyond CSF-based SAA, several other *α*-synuclein biomarkers, including plasma SAA, RT QuIC assays, and PET tracers under development, have been reported, which may further refine the *in vivo* assessment of α-synuclein co-pathology.

#### 3.1.1 Quantitative measurement of α-synuclein in CSF

Quantitative assays of α-synuclein in CSF have been developed as biomarkers reflecting brain α-synuclein accumulation in PD. However, depending on the specific α-synuclein species measured, CSF α-synuclein levels in PD can be either increased or decreased relative to healthy controls, showing considerable inconsistency. The measured concentration is also influenced by disease stage and aggregation state (total versus oligomeric) (Kang et al., 2013; Kapsali et al., 2024). Nonetheless, quantitative CSF *α*-synuclein assays may hold value for monitoring responses to DMTs in PD (Alfaidi et al., 2024).

#### 3.1.2 Assays to measure α-synuclein in blood

Assays leveraging blood-derived neuronal extracellular vesicles or seed amplification techniques have been proposed for the measurement of α-synuclein in blood, although their reproducibility remains a matter of debate (Kluge et al., 2022; Bernhardt et al., 2024). Recently, a novel approach combining immunoprecipitation with seed amplification to selectively capture *α*-synuclein seeds in serum has enabled the identification of distinct synuclein strains across synucleinopathies (Okuzumi et al., 2023). While these findings are promising, large-scale validation and standardization across laboratories are still required before these assays can be implemented in clinical or regulatory settings.

#### 3.1.3 Skin biopsy with fluorescent immunohistochemistry to detect cutaneous phosphorylated *α*-synuclein

Skin biopsy using fluorescent immunohistochemistry to detect phosphorylated α-synuclein in cutaneous nerve fibers has emerged as a promising diagnostic tool. Reported sensitivity and specificity are comparable to those of CSF SAA, and the test correlates with disease severity while being minimally invasive with few complications. Importantly, real-world clinical data indicate a diagnostic change in approximately 65% of patients and a treatment change in 55% following skin biopsy in a tertiary care setting, although a large-scale prospective multicenter validation study has not yet been conducted (Donadio et al., 2021; Gibbons et al., 2024; Isaacson et al., 2024).

#### 3.1.4 Imaging

Structural MRI can serve as a valuable tool for assessing disease progression, as progressive atrophy has been reported in regions such as the putamen and parietal cortex in PD (Berman and Siderowf, 2025). Moreover, because the pattern of structural changes differs among α-synucleinopathies, machine learning approaches applied to structural MRI have demonstrated high sensitivity and specificity in differentiating PD, multiple system atrophy of the parkinsonian type (MSA-P), and PSP (Vaillancourt et al., 2025). Although α-synuclein PET tracer development is advancing, no tracer has yet been validated for routine clinical application (Xiang et al., 2025). Among candidates, [^18^F]-C05-05 has shown binding in the midbrain and striatum of PD and DLB patients, as well as in the putamen and cerebellar peduncle of MSA patients, suggesting that C05-05 interacts with α-synuclein aggregates across different synucleinopathies (Endo et al., 2024). Another tracer, [^18^F]-ACI-12589, demonstrates binding in cerebellar white matter and middle cerebellar peduncles in MSA patients, but not in PD or DLB, indicating potential utility for differential diagnosis among α-synucleinopathies (Smith et al., 2023).

In summary, aside from SAA, most approaches remain insufficiently validated. To date, only SAA have demonstrated high diagnostic accuracy for detecting Lewy body pathology as a co-pathology in AD (Collij et al., 2024b). Further standardization and large-scale validation of other biomarkers are highly anticipated.

### 3.2 Vascular brain injury

Although the 2024 NIA-AA criteria list vascular brain injury (VBI) as a non-AD co-pathology, it does not provide a precise, unified definition. Indeed, multiple definitions exist for vascular cognitive impairment (VCI) (You et al., 2024; Sachdev et al., 2025; Smith et al., 2025; Swartz et al., 2025). In this review, the term VBI broadly encompasses vascular pathology with a spectrum of clinical and subclinical presentations, including cortical and subcortical infarcts, strategic infarcts, small-vessel disease (SVD) with white matter lesions, lacunes, and intracerebral hemorrhages. Despite terminological heterogeneity that complicates epidemiological comparisons, VCI is widely recognized as the second most common cause of dementia, accounting for approximately 20–40% of all cases (Lobo et al., 2000; Kalaria et al., 2008; Chang Wong and Chang Chui, 2022; Rundek et al., 2022; Tuominen et al., 2025).

VBI and AD share aging as a major risk factor and have numerous additional overlapping risk factors (Sato and Morishita, 2015). According to autopsy studies, vascular pathology is about four times more common in AD brains that show mixed pathology than in those with AD pathology alone (Medina-Rioja et al., 2024). Moreover, vascular risk factor burden and AD pathophysiology progress independently yet synergistically, together accelerating neurodegeneration and cognitive decline (Ferrari-Souza et al., 2024). In addition to its relationship with AD, VBI may also interact with TDP-43–related pathology described later in this review, suggesting shared vascular and metabolic vulnerabilities (Grothe et al., 2023; Nelson et al., 2024). Further accumulation of clinicopathological and biomarker evidence will be necessary to clarify whether these associations are causal or simply coexistent.

Among the causes of VCI, SVD represents the major contributor, accounting for approximately 50–70% of cases (Cheng et al., 2024). SVD includes heterogeneous small-vessel pathologies such as arteriolosclerosis and cerebral amyloid angiopathy (CAA). On MRI, SVD is characterized by a constellation of imaging features, including white matter hyperintensities (WMH) and other imaging findings, rather than WMH representing a stand-alone etiologic category. Of note, WMH are increasingly recognized as pathophysiologically heterogeneous lesions and may reflect not only SVD-related vascular injury but also non-vascular mechanisms, including AD-related neurodegenerative processes (Garnier-Crussard et al., 2023). Therefore, Table 1 clarifies the hierarchical relationship among these commonly conflated terms (SVD: disease construct; WMH: imaging marker; and CAA: underlying small-vessel pathology). Hallmarks of SVD include recent small subcortical infarcts, lacunes, white matter hyperintensities (WMH), enlarged perivascular spaces, cerebral microbleeds, cortical superficial siderosis, brain atrophy, and cortical cerebral microinfarcts (Duering et al., 2023). As an AD co-pathology, a higher WMH burden particularly increases the risk of incident dementia and AD (Debette et al., 2019; Duering et al., 2023). Both Aβ pathology and WMH independently impair cognitive performance and synergistically exacerbate future cognitive decline (Ali et al., 2023). Beyond their vascular origin, recent evidence indicates that a subset of WMH in AD may arise from AD-related processes such as tau-mediated axonal degeneration, amyloid toxicity, and neuroinflammation rather than ischemia, highlighting the heterogeneous nature of WMH pathophysiology (Garnier-Crussard et al., 2023). Recognizing this heterogeneity will be essential for interpreting WMH as biomarkers within the AD continuum and for differentiating vascular from degenerative mechanisms in mixed dementias.

**Table 1:** Major vascular pathologies relevant to Alzheimer’s disease.

Vascular pathology	Approximate prevalence in AD	Key imaging markers	Interaction with amyloid and tau
Cerebral small vessel disease (SVD)	Upto 80% in older adults with AD	Recent subcortical infarcts, lacunes, WMH, penvascular space, microbleeds, superficial siderosis, cortical microinfarcts	Vascular pathology contributes to cognitive impairment independently of amyloid and tau
White matter hyperintensities (WMH)	Upto 80% in older adults with clinically diagnosed AD	White matter signal abnormalities of variable size, hyperintense on T2-weighted images without carvitation, assessed by validated visual rating scales (e.g., Fazekas, ARWMC) or manual delineation	WMH are primarily vascular lesions associated with increased dementia risk, including AD; however, amyloid pathology and WMH independently impair cognition and synergistically accelerate cognitive decline, with possible non-vascular contributions to WMH
Cerebral amyloid angiopathy (CAA)	80–100% of AD patients exhibit CAA-like amyloid deposition in intracranial vessels	Strictly lobar haemorrhagic lesions on T2*-weighted MRI, including intracerebral haemorrhage, cerebral microbleeds, cortical superficial, siderosis, and convexity subarachnoid haemorrhage	CAA contributes to cognitive decline through direct vascular dysfunction and indirect interactions with AD pathology, although the underlying mechanisms remain debated

This table provides a conceptual overview of major vascular pathologies relevant to Alzheimer’s disease (AD), including cerebral small vessel disease (SVD), white matter hyperintensities (WMH), and cerebral amyloid angiopathy (CAA). Approximate prevalence estimates in AD cohorts, representative neuroimaging markers, and reported interactions with amyloid and tau pathology are summarized to facilitate cross-entity comparison. Prevalence estimates are approximate and may vary depending on age, disease stage, and cohort characteristics. Descriptions of interactions with amyloid and tau reflect associations reported in the literature and should not be interpreted as evidence of direct causal relationships. Representative pathological and imaging-based evidence supporting these concepts can be found in Attems and Jellinger (2014) and Kao et al. (2019). ARWMC, Age-Related White Matter Changes.

Unlike other forms of VBI, cerebral amyloid angiopathy (CAA) is etiologically related to impaired Aβ processing rather than to traditional systemic vascular risk factors (Wang et al., 2024). Autopsy evidence indicates that 80–100% of AD patient’s exhibit CAA-like amyloid deposition in intracranial vessels, whereas 40–60% of patients with CAA-associated intracerebral hemorrhage show AD-like neuropathological changes (Wang et al., 2024). Pathophysiologically, CAA involves aggregation of Aβ within vessel walls, resulting in vessel fragility (Greenberg et al., 2020). CAA contributes to cognitive decline, although the mechanisms remain under debate. Competing models propose that cognitive impairment arises either directly from CAA-related vascular dysfunction or indirectly through interactions with AD pathology—particularly NFTs (Boyle et al., 2015; Rabin et al., 2022; Godrich et al., 2024; Sin et al., 2024). Clinically, an important complication of CAA is lobar intracerebral hemorrhage, which carries a mortality rate of approximately 10–40% (Wang et al., 2024). CAA is also a recognized risk factor for amyloid-related imaging abnormalities (ARIA), as emphasized in anti-amyloid therapy labels, underscoring the clinical importance of its detection (Hampel et al., 2023; Eisai Inc, 2025; Eli Lilly and Company, 2025b).

In the 2024 NIA-AA criteria, the most definitive vascular biomarkers are anatomic MRI or CT evidence of macroscopic cerebral infarctions; however, no single summary measure yet exists to comprehensively quantify vascular pathology (Jack et al., 2024). Diffusion-weighted imaging is sensitive to SVD-related damage but lacks specificity, and WMH are not unique to SVD (Duering et al., 2023; Silbert et al., 2024). We and others have recently demonstrated that plasma PlGF levels correlate with WMH volume, underscoring its potential role as a surrogate marker for SVD (Igarashi et al., 2025). Continued efforts are underway to standardize and validate new biomarkers and imaging modalities for vascular co-pathologies (Duering et al., 2023).

### 3.3 Limbic-predominant age-related TDP-43 encephalopathy

Limbic-predominant age-related TDP-43 encephalopathy (LATE) is a clinicopathological entity characterized neuropathologically by a stereotypical pattern of TDP-43 proteinopathy, termed LATE neuropathologic change (LATE-NC), and clinically by a slowly progressive amnestic cognitive disorder in older adults that is often difficult to distinguish from AD (Nelson et al., 2019).

It has become increasingly recognized that AD represents only one of several neuropathological diseases associated with amnestic cognitive impairment. In particular, older individuals, especially the oldest-old, may exhibit cognitive decline out of proportion to the severity of ADNC (Schneider et al., 2007; Savva et al., 2009; Nelson et al., 2012). Among the major contributors identified was TDP-43 proteinopathy, now acknowledged as a distinct pathogenic mechanism in late life (Nelson et al., 2013; Josephs et al., 2015). In 2019, a formal consensus first established neuropathologic diagnostic criteria and staging for LATE (Nelson et al., 2019).

Because LATE-NC increases with age, estimates vary across community- or population-based autopsy cohorts with different age-at-death profiles. In a combined analysis of 13 such cohorts, the proportion with LATE-NC ranged from 11.1 to 67.7% (overall ≈39.4%), and cohort mean ages at death spanned 72.2–97.2 years (Nelson et al., 2022). In a strictly population-based study restricted to the oldest-old (Vantaa 85+; all residents ≥85 years), any LATE-NC stage was present in 64.1% of individuals—highlighting how common LATE is at very advanced ages (Mikhailenko et al., 2025). Note that these are autopsy-based frequencies and “do not represent projected population prevalence”. LATE-NC frequently co-occurs with ADNC, affecting more than 50% of those with frequent neuritic plaques (Nelson et al., 2022; Mikhailenko et al., 2025). Such co-pathologies markedly accelerate cognitive decline (Boyle et al., 2017; Katsumata et al., 2020; Montine et al., 2022; Smirnov et al., 2022) and are also associated with memory decline independent of ADNC, which has motivated efforts to develop *in vivo* biomarkers (Nelson et al., 2022; Mikhailenko et al., 2025). With the clinical rollout of anti-amyloid therapies, accurate in vivo diagnosis of LATE has become increasingly important for interpreting DMTs outcomes. However, validated biomarkers for TDP-43 or LATE-NC are currently lacking. Accordingly, proposed clinical diagnostic criteria for LATE incorporate clinical features, imaging findings, and AD biomarker status (Wolk et al., 2025). The criteria suggest that LATE should be considered the primary driver when both core clinical syndrome and a required imaging finding are present. Clinically, this refers to a progressive amnestic syndrome characterized by prominent episodic memory loss, often described as temporo-limbic amnesia, with relative preservation of other cognitive domains, and on imaging to marked hippocampal atrophy that is disproportionate to global atrophy. When LATE is suspected as the primary pathology, a negative amyloid biomarker supports a diagnosis of probable LATE. If amyloid is positive, tau biomarkers are evaluated: a negative tau result indicates possible LATE, whereas a positive tau result necessitates a comprehensive assessment using clinical trajectories and MRI, tau PET, or FDG-PET to determine whether the findings can be explained by AD alone. If not, the diagnosis is possible LATE with AD. In essence, these criteria emphasize the exclusion of findings typical of AD. They serve as an initial framework, and further validation together with the development of specific biomarkers is anticipated.

In research settings, in vivo biomarkers for TDP-43 proteinopathies are under active development, but no disease-specific biomarker for LATE-NC has yet been established. Fluid-based assays face substantial challenges, as TDP-43 is a ubiquitously expressed protein and most currently available antibodies fail to reliably distinguish pathological from physiological species in CSF or plasma. Consequently, measured TDP-43 levels may reflect diverse systemic or neurological injuries rather than LATE-specific pathology. Moreover, the majority of CSF and plasma TDP-43 studies to date have focused on amyotrophic lateral sclerosis or frontotemporal lobar degeneration, limiting their applicability to LATE-NC.

A recent study using a highly sensitive plasma assay demonstrated an association between plasma TDP-43 levels and autopsy-confirmed advanced LATE-NC, particularly in individuals with comorbid AD pathology; however, its ability to detect isolated LATE-NC was less clear, highlighting the current limitations of fluid biomarkers for this condition (Wang et al., 2025b). Although several PET ligands targeting TDP-43 pathology have been proposed at a preclinical or exploratory stage (Vokali et al., 2025), none have yet demonstrated sufficient specificity or validation for clinical in vivo use. Consequently, direct PET-based assessment of TDP-43 pathology in LATE-NC remains unavailable.

In the absence of disease-specific biomarkers, several imaging features have been proposed as supportive indicators of LATE. These include medial temporal and hippocampal atrophy on structural MRI and an elevated inferior temporal-to–medial temporal lobe ratio on FDG-PET, which may help distinguish LATE-related patterns from typical AD trajectories (López-Carbonero et al., 2024).

Finally, under the 2024 NIA-AA criteria, multimodal AT_1_T_2_NISV biomarker profiles can serve as useful indirect indicators of co-pathologies that lack disease-specific biomarkers, such as LATE. In particular, a biomarker profile showing neurodegeneration without tau aggregation (T_2_ − N + profile, also referred to as a T_2_N mismatch) suggests the presence of non-AD injury.

## 4 Resilience and cognitive reserve

### 4.1 Conceptual framework

Individuals whose clinical stage is better than expected for their biological stage are thought to possess exceptional resilience or resistance. Numerous studies have explored the factors that contribute to this phenomenon. However, because different research fields have used overlapping terms with varying definitions, confusion can arise; therefore, we first clarify terminology. Here, terms are organized according to the framework proposed by the National Institute on Aging’s Collaboratory (Stern et al., 2023). *Resilience* is used as an umbrella term defined as “a general term that subsumes any concept that relates to the capacity of the brain to maintain cognition and function with aging and disease.” Within this overarching construct, the framework provides operational definitions for *cognitive reserve* (CR), *brain maintenance* (BM), and *brain reserve* (BR). This concept is illustrated in Figure 2, which integrates biological and clinical staging under the 2024 NIA-AA criteria and depicts how co-pathologies can accelerate, and resilience can delay, clinical progression despite equivalent levels of biological pathology.

### 4.2 Contributing factors and heterogeneity

Building on this conceptual framework, CR refers to individuals who maintain better cognitive performance despite a similar degree of neuropathology. In empirical studies, CR is commonly operationalized using sociobehavioral proxies such as early age IQ, educational attainment, occupational complexity, and cognitively stimulating leisure activities, which modulate the relationship between biomarker burden and clinical expression (Stern, 2012).

BM refers to those whose neuropathology and brain atrophy progress more slowly than that of their peers over time. Genetics and lifestyle can impact BM. For example, APOE genotype modulates vulnerability across the AT(N) cascade, with ε2 conferring relative protection and ε4 increasing susceptibility to amyloid and tau pathology (Tzioras et al., 2019).

BR refers to those who begin life with greater intracranial or neuronal resources and therefore manifest symptoms later in life. Proxies to estimate BR include intracranial volume, head circumference, specific patterns of gray matter volume, and cortical surface area (Stern et al., 2020).

By contrast, *resistance* is defined as “absence or lower than expected levels of AD pathology” (Arenaza-Urquijo and Vemuri, 2020). Accordingly, within the NIA-AA integrated staging system, individuals who present with a better clinical stage than expected for their biological stage can be understood as demonstrating CR within the broader construct of *resilience*.

Many studies have reported that sociobehavioral factors such as education, occupation, and leisure activities, as well as genetic factors such as APOE, contribute to CR (Jagust and Mormino, 2011; Vemuri et al., 2014; Pappalettera et al., 2024). However, because terminology and measurement approaches remain inconsistent, high-quality and reproducible evidence has been limited, emphasizing the need for greater standardization in future research (Pappalettera et al., 2024; Mutel et al., 2025).

Across multiple cohorts applying the 2024 NIA-AA criteria, concordance between biological and clinical staging is estimated at only about 30–50%, demonstrating substantial heterogeneity across the AD continuum (Lu et al., 2024; Mendes et al., 2025; Pichet Binette et al., 2025). This heterogeneity is driven by both co-pathology and resilience. Among these, discordance in which the clinical stage exceeds the biological stage is more common, underscoring the impact of co-pathologies on symptom expression. Conversely, fewer cases show a more advanced biological than clinical stage, likely because tau PET is strongly linked to cognitive decline and clinical progression. These patterns vary depending on cohort composition and the operational definitions used for tau PET–based staging, which are intentionally broad within the 2024 NIA-AA criteria (Mendes et al., 2025; Pichet Binette et al., 2025).

As illustrated in Figure 2, individuals with comparable AT(N) biomarker profiles can occupy markedly different clinical stages, highlighting how resilience-related factors and co-pathologies shape real-world disease trajectories beyond biomarker-defined classifications.

Taken together, these findings indicate that discordance between biological and clinical staging is not an exception but a common feature of AD. Such heterogeneity highlights the limitations of relying solely on biomarker-defined stages to predict clinical outcomes and highlights the importance of incorporating modifiers of reserve and resilience when interpreting disease trajectories. In this context, resilience-related factors provide a conceptual framework for understanding why individuals with comparable AT(N) profiles may follow divergent clinical courses.

## 5 Disease-modifying therapies

At present, two DMTs are in clinical use worldwide: the anti-amyloid monoclonal antibodies lecanemab-irmb (Leqembi) and donanemab-azbt (Kisunla).

### 5.1 Lecanemab

In the United States, lecanemab received accelerated approval from FDA in 2023 and, following the results of the phase 3 trial, became the first DMT for AD to receive full FDA approval (U.S. Food and Drug Administration, 2023b; van Dyck et al., 2023; Eisai Inc, 2025). After full approval, the therapy was also covered by Medicare, enabled broad clinical implementation (Centers for Medicare and Medicaid Services, 2023b). The indication is consistent across FDA labeling and Medicare coverage and aligns with the inclusion criteria used in clinical trials, specifically targeting patients in the MCI or mild dementia stage. Before initiating therapy, the FDA labeling specifies that “the presence of amyloid beta pathology must be confirmed prior to initiating treatment,” and Medicare likewise states that “your doctor or other health care provider must confirm you have beta-amyloid plaques consistent with Alzheimer’s disease,” without specifying a particular diagnostic modality (U.S. Food and Drug Administration, 2023b; Centers for Medicare and Medicaid Services, 2025). During treatment, brain MRI is required to monitor for ARIA.

Japan followed the United States in approving lecanemab and listing it for National Health Insurance reimbursement in 2023 (Eisai Co., Ltd, 2023). Japan’s optimal-use guideline explicitly requires confirmation of amyloid pathology by amyloid PET or CSF (Ministry of Health, LAW, 2023a). MCI or mild dementia due to AD, defined using both the Mini-Mental State Examination (MMSE) and the Clinical Dementia Rating (CDR 0.5–1). For safety monitoring, in addition to the FDA-recommended MRI scans before the 5th, 7th, and 14th doses, MRI every 6 months thereafter is recommended.

In the European Union (EU), lecanemab initially received a negative opinion but was later authorized by the European Commission in April 2025 following re-examination. However, because of the risk of ARIA, the indication is restricted to patients with early-stage AD who carry either zero (non-carriers) or one copy (heterozygous carriers) of the APOE ε4 allele (European Medicines Agency, 2025b).

### 5.2 Donanemab

Donanemab is a novel anti-amyloid monoclonal antibody designed for administration over a fixed treatment period (Sims et al., 2023). Whereas lecanemab primarily targets soluble Aβ aggregates (protofibrils), donanemab targets pyroglutamate-modified amyloid beta at the third amino acid position (N3pG-Aβ), which is believed to exist only within insoluble amyloid plaques.

Donanemab was approved by FDA in 2024 and subsequently covered by Medicare. Its indication mirrors that of lecanemab, targeting patients with MCI or mild dementia due to AD, and includes requirements for regular MRI monitoring (Centers for Medicare and Medicaid Services, 2022; Eli Lilly and Company, 2025b). The therapy was listed for National Health Insurance reimbursement in Japan in 2024 (Eli Lilly Japan KK, 2024; Ministry of Health, LAWJ 2025b). In the EU, donanemab received authorization from the European Commission in September 2025, with its indication similarly restricted to APOE ε4 non-carriers or heterozygous carriers (European Medicines Agency, 2025a). Pricing and reimbursement are determined separately by each member state’s national health authorities.

### 5.3 Comparative insights

Lecanemab and donanemab are both anti-amyloid monoclonal antibodies approved for early AD. The key distinction lies in their targets: lecanemab binds soluble amyloid beta protofibrils, whereas donanemab recognizes pyroglutamate-modified amyloid beta within insoluble plaques. Both require confirmation of amyloid pathology and regular MRI monitoring for ARIA, but donanemab is designed for a fixed-duration course, while lecanemab is administered continuously. Because ARIA risk is higher in APOE ε4 homozygotes, the EU restricts treatment to non-carriers and heterozygotes. Overall, both agents mark the beginning of biomarker-guided precision therapy in AD.

It should be noted that no head-to-head clinical trials directly comparing lecanemab and donanemab have been conducted to date. As a result, differences in efficacy and safety between these agents are inferred from separate trials with distinct designs, inclusion criteria, and outcome measures, and should be interpreted with caution. Moreover, post-approval experience has highlighted that the incidence, management, and practical impact of ARIA may differ in real-world settings compared with rates reported in pivotal trials. For example, early real-world data from Japan indicate that while treatment discontinuation due to ARIA has been relatively infrequent, substantial logistical and infrastructural challenges remain, including MRI capacity, infusion resources, and access to APOE genotyping, highlighting the complexity of implementing disease-modifying therapies beyond regulatory trial settings (Sato et al., 2018).

## 6 Implementation of biomarkers in clinical practice

**Figure 3:**
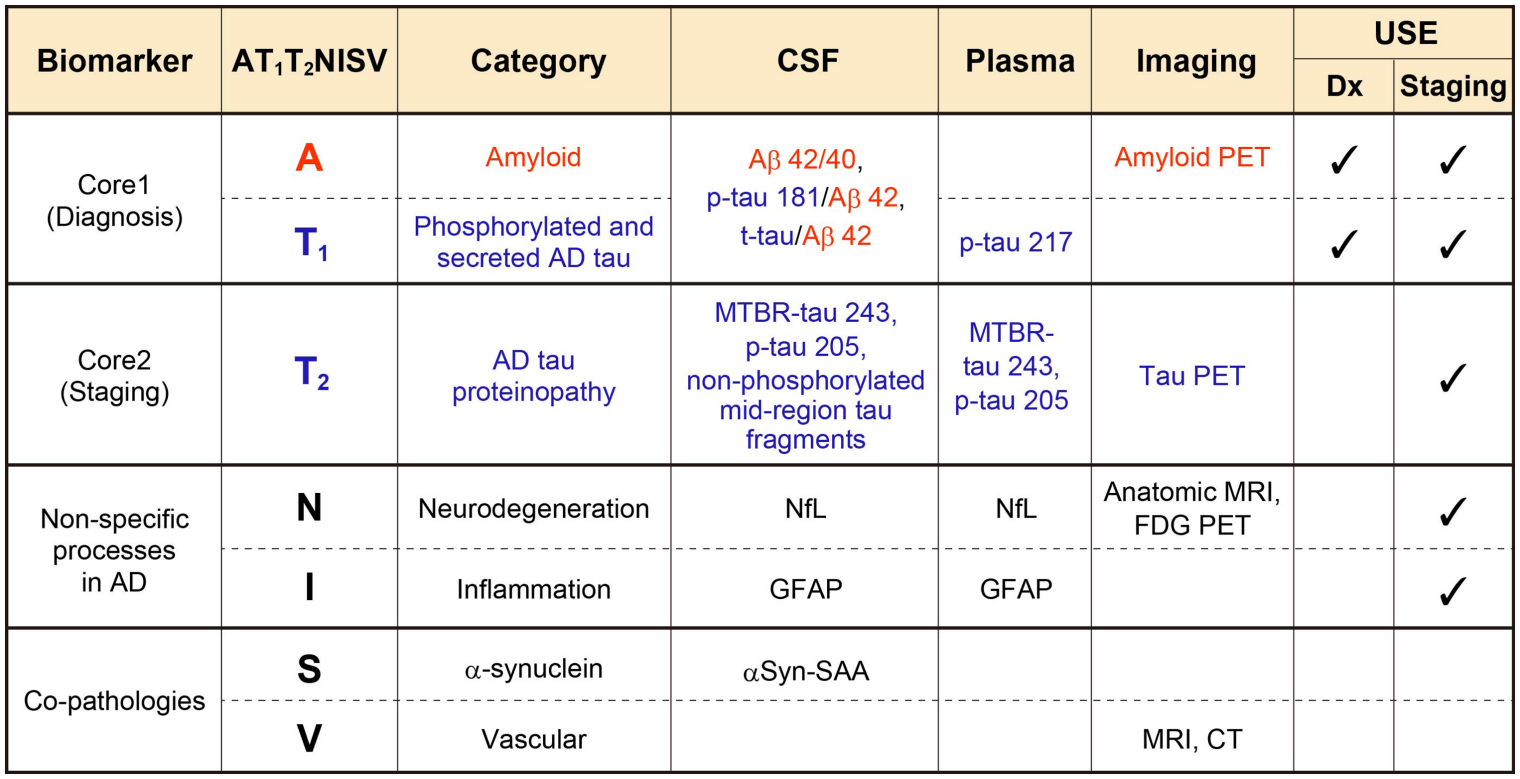
Biomarker categories and representative assays in the 2024 NIA-AA framework. Core 1 biomarkers (A, T₁) define biological AD and are used for diagnosis, whereas Core 2 biomarkers (T₂) are used for biological staging. Additional biomarkers (N, I, S, V) represent nonspecific processes or co-pathologies, including neurodegeneration (NfL, MRI, FDG PET), inflammation (GFAP), α-synuclein (αSyn-SAA), and vascular injury (MRI, CT). Corresponding CSF, plasma, and imaging modalities for each biomarker class are shown. Concept adapted from Jack et al. (2024), Alzheimer’s and Dementia.

The 2024 NIA-AA criteria established an integrated framework that classifies biomarkers into six categories—A, T₁, T₂, N, I, S, and V—each reflecting distinct biological processes and clinical implications. Core 1 biomarkers (A and T₁) define biological AD and are required for diagnosis, while Core 2 biomarkers (T₂) provide information for staging. Additional categories capture nonspecific processes or co-pathologies, including neurodegeneration (NfL, MRI, FDG PET), inflammation (GFAP), *α*-synuclein (αSyn-SAA), and vascular injury (MRI, CT). These relationships are summarized in Figure 3, which outlines representative imaging, CSF, and plasma assays corresponding to each biomarker class within the 2024 NIA-AA criteria.

### 6.1 Imaging biomarkers

#### 6.1.1 Amyloid PET

In the United States, three fluorine-18–labeled amyloid radiotracers (^18^F-florbetapir, ^18^F-flutemetamol, and ^18^F-florbetaben) are currently FDA-approved as an amyloid PET tracers. Initially, their indication was “evaluation of Alzheimer’s disease (AD) and other causes of cognitive decline,” but following label updates in 2025, the indication was expanded to include “selection of patients who are candidates for amyloid beta-directed therapy”.

In clinical practice, amyloid PET images are generally evaluated using a qualitative visual read, and regulatory approvals have been based primarily on this approach. Each tracer has its own standardized reading criteria, and scans are classified binarily as positive or negative (Chapleau et al., 2022). The Imaging Dementia–Evidence for Amyloid Scanning (IDEAS) study, conducted under Medicare to evaluate the clinical utility of visually interpreted amyloid PET in patients with MCI or dementia, reported management change in roughly two-thirds of cases after the scan, and 4.49% relative reduction in 12-month hospitalization versus matched control (though it fell short of the prespecified effect size) (Rabinovici et al., 2019, 2023).

In Japan, following the 2023 approval of lecanemab, amyloid PET was listed for national reimbursement as a diagnostic test required for patient selection prior to treatment initiation (Ministry of Health, LAWJ 2025c). In 2024, with the approval of donanemab, “confirmation of amyloid beta plaque removal by anti-amyloid beta antibody therapy” was also added as a reimbursable indication. According to the *Appropriate Use Guideline for Amyloid PET Imaging Agents* (Japanese Society of Nuclear Medicine, 2025) image interpretation is based on binary visual assessment. When quantitative values are referenced, visual interpretation should remain the primary method, with appropriate consideration of the limitations of quantitative measures.

In the EU, amyloid PET tracers are also authorized, although reimbursement in clinical practice varies by country (European Medicines Agency, 2017, 2018, 2019). The European Medicines Agency (EMA) has issued a qualification opinion recognizing the CL scale as a useful adjunct to visual reading for quantitative assessment.

Within the EU regulatory framework, the EMA’s Committee for Medicinal Products for Human Use (CHMP) conducts the scientific evaluation of medicines, and the European Commission grants the legally binding EU-wide marketing authorization. This centralized authorization is valid across all Member States. However, pricing and reimbursement are not determined by the EMA; they are decided separately by each country’s national health authorities, as is the case for disease-modifying therapies.

It is important to note that visual read remains the primary interpretive method in current practice, whereas quantitative metrics serve as useful adjuncts that enhance consistency, comparability and longitudinal evaluation.

#### 6.1.2 Tau PET and coverage policies

In 2020, the tau radiotracer FTP was approved by FDA “to estimate the density and distribution of neurofibrillary tangles (NFTs) in adult patients with cognitive impairment who are being evaluated for AD” (Avid Radiopharmaceuticals, 2024).

Regarding Medicare policy, when amyloid PET was first covered in 2013, reimbursement was restricted under Coverage with Evidence Development (CED), a mechanism allowing Medicare coverage of a diagnostic or therapy contingent upon participation in a clinical trial or registry to assess effectiveness. Coverage was limited to a single lifetime scan and to two specific indications: (1) to exclude AD in narrowly defined and clinically challenging differential diagnoses, such as AD versus frontotemporal dementia (FTD); and (2) to enrich AD clinical trials by selecting participants based on biological, clinical, and epidemiological factors (Centers for Medicare and Medicaid Services, 2013). However, in 2023, with the emergence of DMTs and growing evidence for the clinical utility of amyloid PET, Centers for Medicare and Medicaid Services (CMS) decided to remove both the CED requirement and the once-in-a-lifetime restriction, expanding access for clinical use (Centers for Medicare and Medicaid Services, 2023a).

#### 6.1.3 PET standardization for clinical implementation

Visual interpretation is inherently operator-dependent, and discrepancies in reader training or experience can lead to inconsistent results across sites. Moreover, because amyloid accumulation represents a continuous biological process, binary classification alone may not fully capture the disease spectrum. Therefore, several quantitative approaches have been proposed to improve comparability and sensitivity (Pemberton et al., 2022).

Among these, the Centiloid (CL) scale was introduced to standardize amyloid PET signals across different tracers and analysis pipelines, providing a tracer-independent metric for quantitative comparison (Klunk et al., 2015; Collij et al., 2024a; European Medicines Agency, 2024). The CL scale uses two anchor points—0 CL (mean signal in cognitively unimpaired individuals ≤45 years) and 100 CL (mean signal in typical mild-to-moderate AD)—to normalize amyloid PET data. Although now widely adopted, the CL value can be inaccurate in certain cases, such as when cerebellar reference signal is abnormally high or when off-target uptake occurs outside predefined volume of interests (VOIs) (Collij et al., 2024a). In the IDEAS study, agreement between visual reads and quantitative classification (>24.4 CL) was 86.3% (Zeltzer et al., 2025).

In practice, the CL scale can be applied for (i) population characterization, (ii) participant selection (eligibility criteria), and (iii) treatment monitoring, such as verifying amyloid removal or clearance in trials of amyloid-targeting therapies (Iaccarino et al., 2025). For population characterization, CL values correlate with overall amyloid burden and help position individuals along the AD continuum. CL < 10 effectively excludes neuritic amyloid pathology, CL > 20 supports the presence of at least moderate neuritic plaques, and CL > 50 is strongly consistent with a neuropathological diagnosis of AD (Amadoru et al., 2020; Pemberton et al., 2022). For treatment monitoring, reductions in CL values have been used to confirm amyloid clearance and to compare pharmacodynamic effects across DMTs (Mintun et al., 2021; Van Dyck et al., 2023). Reflecting these advances, the revised FDA labels now state that “quantification of amyloid beta neuritic plaque levels [e.g., CL scale or standardized uptake value ratio (SUVR)] can be used in conjunction with visual assessment,” thereby acknowledging the clinical utility of quantitative evaluation (Eli Lilly and Company, 2025a; GE Healthcare, Medi-Physics, Inc, 2025; Life Molecular Imaging Ltd, 2025).

As with amyloid PET, tau PET is clinically assessed by visual interpretation, although inter-reader variability remains a challenge (Avid Radiopharmaceuticals, 2024). In research settings, quantitative analysis using the SUVR—the ratio of tracer uptake in a target region to a relatively pathology-free reference region—is increasingly used to enhance reproducibility and comparability. In analogy to the CL scale for amyloid PET, recent efforts have led to the development of a universal quantitative framework for tau PET, termed the CenTauR scale (Villemagne et al., 2023; Leuzy et al., 2024). The CenTauR framework provides standardized cortical masks and a harmonized tau PET scale (CenTauRz) that integrates quantitative measures across six major tracers—^18^F-flortaucipir, ^18^F-MK6240, ^18^F-PI2620, ^18^F-PM-PBB3, ^18^F-RO948, and ^18^F-GTP1—and across different processing pipelines. By accounting for tracer-specific characteristics and excluding off-target regions, CenTauR enables robust cross-tracer and cross-center comparison of tau burden and improves discrimination between high and low tau load. This framework represents a major step toward universal standardization of tau PET quantification, supporting multicenter studies and longitudinal monitoring of disease progression.

Quantitative tau PET offers several advantages: (i) it can identify individuals with intermediate NFTs burden, reflecting early-stage disease (Kotari et al., 2023); (ii) it can predict cognitive decline and cortical atrophy quantitatively (La Joie et al., 2020; Biel et al., 2021); and (iii) it facilitates evaluation of longitudinal progression and therapeutic response (Edwards et al., 2023). Beyond staging, the ability to detect and quantify tau pathology has greatly improved the diagnostic accuracy of AD, a concept further elaborated in the section describing the 2024 NIA–AA criteria.

Taken together, Centiloid and CenTauR should be viewed as complementary frameworks—Centiloid providing a mature and widely implemented standard for amyloid PET, and CenTauR addressing the greater biological and tracer heterogeneity inherent to tau imaging.

### 6.2 Fluid biomarkers

#### 6.2.1 The United States

In the United States, FDA has authorized several CSF *in vitro* diagnostic (IVD) assays for AD pathology assessment, including Lumipulse G Aβ42/40 and the Elecsys CSF ratios p-tau181/Aβ42 and t-tau/Aβ42 (U.S. Food and Drug Administration, 2022a; U.S. Food and Drug Administration, 2022b; U.S. Food and Drug Administration, 2023a). In 2025, the first FDA-authorized blood test, the Lumipulse G p-tau217/Aβ42 plasma ratio, was approved to aid in identifying amyloid pathology in adults with cognitive symptoms (U.S. Food and Drug Administration, 2025). Later in 2025, Roche’s Elecsys p-tau181 assay was authorized for primary-care rule-out of amyloid pathology related to AD, (Roche Diagnostics, 2025). Medicare coverage is independent of FDA authorization. In the absence of a National Coverage Determination, reimbursement decisions are made by local Medicare Administrative Contractors under the “reasonable and necessary” standard.

#### 6.2.2 Japan

In Japan, the CSF Aβ42/40 and CSF p-tau181/Aβ42 ratios are approved and reimbursed for confirming amyloid beta pathology when determining eligibility for anti-amyloid therapies in patients with MCI or mild dementia due to AD, as well as in specific treatment restart situations (Ministry of Health, LAWJ 2023c; Ministry of Health, LAW 2025a). CSF p-tau181 is also approved and reimbursed for the differential diagnosis of dementia. Following the approval of lecanemab, concurrent billing for multiple CSF biomarkers, or for CSF biomarkers together with amyloid PET, was not permitted. However, after the approval of donanemab, concurrent billing for CSF biomarkers and amyloid PET became available (Ministry of Health, LAWJ 2023b; Ministry of Health, LAWJ 2024). Plasma Aβ1-42 and Aβ1-40 assays are approved as IVDs but are not reimbursed. According to the *Appropriate Use Guideline for CSF and Blood Biomarkers for Dementia* (Guideline Development Committee, 2025), plasma p-tau217 and the p-tau217/Aβ42 ratio demonstrate higher diagnostic accuracy than plasma Aβ42/40 and should be used as pre-screening tools prior to CSF or PET testing for anti-amyloid therapy. Stand-alone use of plasma biomarkers to directly determine treatment eligibility is not recommended, and they are either not approved as IVDs or covered by national insurance at this time.

#### 6.2.3 European union

In the EU, IVDs are not approved by EMA. Instead, manufacturers obtain a CE mark under IVDR—often via a Notified Body—which allows marketing across the European Economic Area (EEA). Whether IVDs are reimbursed is decided country by country. Currently, multiple IVDs are CE marked, which measure Aβ42, Aβ42/40, p-tau181, t-tau, Aβ42/p-tau181, p-tau181/Aβ42 in CSF, and Aβ42/40, p-tau181 in plasma (Iaccarino et al., 2023; F. Hoffmann-La Roche Ltd, 2025).

### 6.3 Discussion and future perspectives

#### 6.3.1 Discussion

The 2024 NIA-AA criteria have established a biological definition of AD based on Aβ and tau biomarkers (Jack et al., 2024). This represents a major conceptual advance that unifies research and clinical approaches under a biological paradigm. Nevertheless, increasing evidence indicates that Aβ and tau capture only part of the disease biology, while age-related vulnerability factors—vascular, glial, and metabolic—also shape disease expression and progression (Nelson et al., 2024). Thus, the current framework should be viewed as a foundation for understanding dementia biology rather than a final model.

Although Aβ positivity defines the biological entry point of AD, Aβ deposition alone does not always reflect the full neuropathologic spectrum of AD. Some Aβ-positive individuals exhibit additional age-related pathologies such as LATE-NC (Nelson et al., 2019), argyrophilic grain disease (AGD) with incidental Aβ, or mixed proteinopathies. Confirming tau pathology (T^+^) through PET or fluid biomarkers can therefore refine diagnostic specificity and distinguish AD defined by both Aβ and tau from age-related amyloidosis. Likewise, co-pathologies involving TDP-43, *α*-synuclein, and vascular brain injury may influence clinical trajectories and therapeutic response, highlighting the need for biomarker-based stratification beyond Aβ status when assessing DMTs.

At the same time, Aβ-negative dementia remains a major diagnostic and conceptual challenge. Such cases may represent LATE-NC, primary age-related tauopathy (PART), DLB, or early AD below current detection thresholds. Although no validated fluid biomarker for TDP-43 currently exists, recent preprint studies have proposed plasma-based assays and reported encouraging preliminary findings suggesting that peripheral TDP-43 signatures may reflect brain pathology (Wang et al., 2025a). Likewise, emerging imaging observations, such as regional mismatches between tau deposition and medial temporal atrophy may offer indirect clues to identify LATE-NC *in vivo* (Lyu et al., 2025). Integrating these experimental approaches into the existing biological framework could, in the future, extend the diagnostic reach of the 2024 NIA-AA criteria and enable more precise delineation of amnestic disorders beyond the canonical Aβ–tau continuum.

Mechanistically, Aβ and tau accumulation not only induce synaptic dysfunction but also disrupt the neurovascular unit, astrocytic metabolism, and glymphatic clearance, thereby accelerating brain aging (Thal et al., 2025). Within this stressed milieu, TDP-43, normally a nuclear RNA-binding protein, may undergo cytoplasmic aggregation, resulting in RNA dysregulation, mitochondrial damage, and neuronal death (Nelson et al., 2023; Sharma et al., 2024). Rather than a competing entity, such TDP-43 pathology can be viewed as a downstream manifestation within a shared vulnerability axis linking proteinopathy, vascular integrity, and glial metabolism. Postmortem and neuroimaging studies support this interpretation: hippocampal atrophy is mild in “pure” AD (TDP-43–negative, no vascular disease) but more pronounced in cases with TDP-43 or vascular co-pathology (Gunasekaran et al., 2025). In this context, hippocampal atrophy may be better understood not solely as a marker of AD severity but as an indicator of cumulative pathological burden.

Clinically, while anti-amyloid therapies have introduced a new era of DMTs, many Aβ-positive patients remain ineligible because of age, comorbidities, or high risk of ARIA (brain MRI and APOE status). This underscores the urgent need for alternative DMTs targeting tau, inflammation, or metabolic dysfunction. Conversely, patients with Aβ-negative amnestic syndromes, potentially representing LATE-NC or other pathologies, currently lack disease-specific treatment options, and evidence-based guidance for management remains limited. Furthermore, the increasing use of biomarkers for identifying “asymptomatic biological AD” raises ethical and psychosocial considerations. Clear protocols for disclosure, counseling, and longitudinal monitoring will be essential to prevent overdiagnosis and to promote informed decision-making.

#### 6.3.2 Future perspectives

Future frameworks should move beyond binary biomarker definitions to capture the quantitative and multifactorial nature of neurodegeneration. Incorporating measures of co-pathologies and age-related vulnerabilities, and identifying the predominant drivers of neurodegeneration in each individual, will be essential for advancing precision medicine in dementia. Multimodal integration of fluid and imaging biomarkers—PET for spatial resolution, plasma and CSF assays for sensitivity and accessibility, and functional or metabolic MRI such as magnetic resonance spectroscopy (Hirata et al., 2023) and perfusion imaging using arterial spin labeling for early astrovascular alterations—will bridge molecular pathology with systems-level dysfunction and guide early intervention strategies.

Ultimately, the biological definition of AD should serve as a flexible framework—one that can evolve to encompass vascular, glial, and TDP-43–related mechanisms—rather than a rigid diagnostic boundary. Translating this evolving biological understanding into clinical practice will require not only technological innovation but also ethical guidance, policy alignment, and patient-centered communication strategies to realize the promise of personalized, biomarker-based medicine for the aging brain.

An important conceptual implication of the biological definition of AD is the re-evaluation of structural neurodegeneration markers traditionally used in clinical diagnosis. In the 2011 NIA-AA criteria (McKhann et al., 2011), medial temporal lobe atrophy was incorporated as a core feature of AD-related neurodegeneration in research frameworks. However, the 2018 ATN framework (Jack et al., 2018) deliberately repositioned neurodegeneration (N) as a marker of disease severity rather than a defining diagnostic element, and subsequent recommendations have increasingly emphasized biomarker-based classification. Despite this conceptual shift, hippocampal atrophy continues to be widely interpreted in clinical practice as a primary indicator of AD. Emerging evidence suggests that hippocampal atrophy is not specific to amyloid or tau pathology and may instead predominantly reflect co-pathological processes that increase with aging, such as LATE-NC or AGD.

In contrast, recent long-term longitudinal studies indicate that structural alterations in white matter and ventricular enlargement precede hippocampal atrophy by several years, even among amyloid-positive individuals (Uchida et al., 2024). These findings raise the hypothesis that imaging markers beyond the medial temporal lobe, particularly white matter–related measures, may provide greater sensitivity for capturing early neurodegenerative processes within the biological framework of AD. Re-aligning the interpretation of structural imaging biomarkers with this evolving biological understanding will be critical for avoiding diagnostic ambiguity and for guiding future biomarker-driven clinical strategies.
